# Accuracy of conventional and novel scoring systems in predicting severity and outcomes of acute pancreatitis: a retrospective study

**DOI:** 10.1186/s12944-021-01470-4

**Published:** 2021-04-27

**Authors:** Qing Wu, Jie Wang, Mengbin Qin, Huiying Yang, Zhihai Liang, Guodu Tang

**Affiliations:** 1 Department of Gastroenterology, the Second Affiliated Hospital of Guangxi Medical University, Nanning, China; 2 Department of Gastroenterology, the First Affiliated Hospital of Guangxi Medical University, Nanning, China

**Keywords:** Acute pancreatitis, Severity, Acute respiratory distress syndrome, Acute renal failure, Mortality, Scoring system, Predict, Retrospective

## Abstract

**Background:**

Recently, several novel scoring systems have been developed to evaluate the severity and outcomes of acute pancreatitis. This study aimed to compare the effectiveness of novel and conventional scoring systems in predicting the severity and outcomes of acute pancreatitis.

**Methods:**

Patients treated between January 2003 and August 2020 were reviewed. The Ranson score (RS), Glasgow score (GS), bedside index of severity in acute pancreatitis (BISAP), pancreatic activity scoring system (PASS), and Chinese simple scoring system (CSSS) were determined within 48 h after admission. Multivariate logistic regression was used for severity, mortality, and organ failure prediction. Optimum cutoffs were identified using receiver operating characteristic curve analysis.

**Results:**

A total of 1848 patients were included. The areas under the curve (AUCs) of RS, GS, BISAP, PASS, and CSSS for severity prediction were 0.861, 0.865, 0.829, 0.778, and 0.816, respectively. The corresponding AUCs for mortality prediction were 0.693, 0.736, 0.789, 0.858, and 0.759. The corresponding AUCs for acute respiratory distress syndrome prediction were 0.745, 0.784, 0.834, 0.936, and 0.820. Finally, the corresponding AUCs for acute renal failure prediction were 0.707, 0.734, 0.781, 0.868, and 0.816.

**Conclusions:**

RS and GS predicted severity better than they predicted mortality and organ failure, while PASS predicted mortality and organ failure better. BISAP and CSSS performed equally well in severity and outcome predictions.

## Background

Acute pancreatitis (AP) is an inflammatory disease of the pancreas with a worldwide incidence varying from 33.2/100,000 to 45/100,000 in the general population [1–3]. Approximately 10% ~ 20% of patients with AP have a severe clinical course, with significant morbidity and mortality due to local and systemic complications [3–6]. Acute respiratory distress syndrome (ARDS) and acute renal failure (ARF) are common complications of severe acute pancreatitis, and result in worse outcomes [7–9]. Therefore, the early detection of ARDS and ARF in patients with AP is indispensable.

Many studies have compared biochemical markers and various scoring systems in the early stage to predict disease course and outcomes in AP [10–13]. Conventional scoring systems, including the Ranson score (RS), Glasgow score (GS), and Acute Physiology, Chronic Health Evaluation (APACHE) II score, and bedside index of severity in acute pancreatitis (BISAP) have been used to assess the severity of AP. However, these scores are complicated and require multiple difficult clinical parameters for risk stratification. Although biomarkers are easy to obtain, their ability in predicting outcomes varies [14–17]. Recently, some novel scoring systems have been developed. A prospective cohort study [18] showed that the pancreatic activity scoring system (PASS; Table 1), which was first reported by the Southern California Pancreas Study Group in 2017 [19], could predict important clinical events at different points during the course of AP. Another new scoring system called the Chinese simple scoring system (CSSS; Table 2) was proposed in 2020 [20]. Both scoring systems are not yet widely used.

**Table 1:** Pancreatic activity scoring system (PASS)

Parameter	Weights
Organ failure	× 100 for each system
SIRS	× 25 for each criterion
Abdominal pain (0–10)	× 5
Morphine equivalent dose (mg)	× 5
Tolerating solid diet (yes = 0, no = 1)	× 40

*SIRS* Systemic inflammatory response syndrome; Organ failure definition: modified Marshall or SOFA score ≥ 2 points in any category

**Table 2:** Chinese simple scoring system (CSSS)

Variables	0	1	2	3	4
Serum creatinine (μmol/L)	< 100	>100			
Blood glucose (mmol/L)	< 12	>12			
LDH (U/L)	< 380	>380			
CRP (mmol/L)	< 65	>65			
Heart rate (beats/min)	< 100	>100			
Extent of pancreatic necrosis	0	< 30%	30–50%	50–70%	>70%

*LDH* Lactate dehydrogenase, *CRP* C-reactive protein

The present study aimed to specifically determine the accuracy of these conventional and novel scoring systems as well as biomarkers in predicting severity, mortality, and organ failure in patients with AP.

## Materials and methods

### Study design and patient selection

A retrospective study was conducted. Records of patients with AP who were treated in The First Affiliated Hospital of Guangxi Medical University, between January 2003 and July 2020, were reviewed.

Patients were diagnosed with AP if they met at least two of the following three criteria: (1) abdominal pain consistent with AP, (2) serum lipase activity or amylase activity at least three times greater than the upper limit of normal, and (3) characteristic findings on abdominal imaging. Patients younger than 16 years, those known to have chronic pancreatitis, or those without sufficient data were excluded from the study.

### Definitions of severity and organ failure

Severity of AP was evaluated based on the revised Atlanta classification [21]. Mild AP was defined as AP in the absence of organ failure and local/systemic complications. Severe AP was characterized by the presence of organ failure and/or local complications. Organ failure was defined according to the modified Marshall scoring system [22].

### Biochemical markers, scoring systems, and their cutoffs

Biochemical markers measured within 48 h after admission were analysed. RS [23], GS [24], BISAP [25], PASS [19], and CSSS [20] were calculated for each patient within 48 h after admission. Scores were compared for their accuracy in the prediction of disease severity, mortality, and development of organ failure (ARDS and ARF).

### Statistical analysis

SPSS v23.0 (IBM Corp., Armonk, NY) was used for statistical analyses. Continuous variables were displayed as mean ± standard deviation. The Student t-test was used for continuous variables. The chi-square test was used for categorical variables. Univariate and multivariate logistic regression analyses were carried out to identify risk factors. Potential risk factors with *P* < 0.05 in the univariate analyses were enrolled into the binary logistic backward stepwise regression analysis. The results are presented as odds ratios (OR) with 95% confidence intervals (CIs). ROC curves of the scores were used for the prediction of severe AP, mortality, ARDS, and ARF. Areas under the curve (AUCs) were used to evaluate the predictive accuracy of each scoring system. All optimum cutoffs were identified on the basis of the highest sensitivity and specificity values generated from the ROC curves. Sensitivity, specificity, positive predictive value (PPV), and negative predictive value (NPV) were calculated. A *P* value of less than 0.05 was considered to indicate statistical significance.

## Results

### Baseline characteristics

Among 1848 patients enrolled, 1164 (62.99%) had mild AP and 684 (37.01%) had severe AP. The mean age of the patients was 48.22 ± 16.21 years. The mean age of severe group was significantly higher in the severe AP group than in the mild AP group (*P* < 0.001). A male preponderance (68.19%) was found. ARF was more common in male patients than in female patients (*P* < 0.001). A higher body-mass index (BMI) was observed in the severe AP group than in the mild AP group (*P* < 0.001). The BMI of patients with ARDS/ARF was higher than those of patients without ARDS/ARF (*P* < 0.05; Table 3). Gallstones (38.47%) were the most common cause of AP, followed by hypertriglyceridemia (16.72%) and alcohol consumption (10.77%). Alcohol-associated pancreatitis was more common in the severe AP group, ARDS group, and ARF group (Table 3). Hyperlipidemia (14.88%) and type-2 diabetes mellitus (7.52%) were common comorbidities. A history of smoking and alcohol intake history was present in 541 (29.27%) and 591 (31.98%) patients, respectively. Alcohol consumption was more common in patients with severe AP (*P* < 0.001), ARDS (*P* = 0.002), and ARF (*P* < 0.001; Table 3). Longer hospital stay was observed in patients with severe AP than in patients with mild AP (*P* < 0.001). The mortality rate was much higher in the severe AP group than in the mild AP group (*P* < 0.001; Table 3).

**Table 3:** Univariate analysis of factors associated with severity, mortality, ARDS, and ARF in AP

Characteristic	Severity			Mortality			ARDS			ARF		
	Mild (*n* = 1164)	Severe (*n* = 684)	*P*	Survivor (*n* = 1782)	Non-survivor (*n* = 66)	*P*	No (*n* = 1735)	Yes (*n* = 113)	*P*	No (*n* = 1706)	Yes (*n* = 142)	*P*
Age, y	46.22 (15.40)	51.62 (16.99)	< 0.001	48.07 (16.10)	52.09 (18.69)	0.048	48.12 (16.18)	49.79 (16.66)	0.288	48.12 (16.22)	49.41 (16.12)	0.363
Male gender, n (%)	783 (67.27)	477 (69.74)	0.271	1210 (67.90)	50 (75.76)	0.178	1175 (67.72)	85 (75.22)	0.097	1142 (66.94)	118 (83.10)	< 0.001
BMI, kg/m^2^	23.43 (4.26)	24.73 (4.52)	< 0.001	23.99 (4.45)	23.07 (2.92)	0.293	23.85 (4.39)	25.44 (4.44)	0.009	23.85 (4.33)	25.51 (5.16)	0.006
Comorbidities, n (%)												
Hyperlipidemia	169 (14.52)	106 (15.50)	0.568	267 (14.98)	8 (12.12)	0.521	257 (14.81)	18 (15.93)	0.747	245 (14.36)	30 (21.13)	0.03
T2DM	85 (7.30)	54 (7.89)	0.641	134 (7.52)	5 (7.58)	0.986	130 (7.49)	9 (7.96)	0.854	135 (7.91)	4 (2.82)	0.013
Etiology, n (%)												
Gallstones	467 (40.12)	244 (35.67)	0.058	697 (39.11)	14 (21.21)	0.003	681 (39.25)	30 (26.55)	0.007	679 (39.80)	32 (22.54)	< 0.001
Alcoholism	101 (8.68)	98 (14.33)	< 0.001	191 (10.72)	8 (12.12)	0.718	178 (10.26)	21 (18.58)	0.006	169 (9.91)	30 (21.13)	< 0.001
Hypertriglyceridemia	186 (15.98)	123 (17.98)	0.265	301 (16.89)	11 (16.67)	0.308	288 (16.60)	21 (18.58)	0.584	278 (16.30)	31 (21.83)	0.089
Smoker, n (%)	337 (28.95)	204 (29.82)	0.691	525 (29.46)	16 (24.24)	0.36	505 (29.11)	36 (31.86)	0.533	490 (28.72)	51 (35.92)	0.07
Alcohol intake history, n (%)	334 (28.69)	257 (37.57)	< 0.001	568 (31.87)	23 (34.85)	0.611	540 (31.12)	51 (45.13)	0.002	520 (30.48)	71 (50.00)	< 0.001
Hospital stay, d	12.35 (8.18)	16.56 (11.95)	< 0.001	14.01 (9.68)	10.08 (14.82)	0.042	13.53 (8.85)	19.38 (19.8)	0.003	13.58 (9.02)	17.52 (17.16)	0.009
Death, n (%)	6 (0.52)	60 (8.77)	< 0.001	–	–	–	29 (1.67)	37 (32.74)	< 0.001	26 (1.52)	40 (28.17)	< 0.001
WBC (*10^9^/L)	10.46 (4.96)	14.71 (6.37)	< 0.001	11.94 (5.77)	14.58 (8.26)	0.012	11.84 (5.81)	14.89 (6.36)	< 0.001	11.79 (5.58)	14.92 (8.28)	< 0.001
Hemoglobin (g/L)	127.61 (21.99)	128.11 (30.90)	0.713	128.26 (24.63)	115.28 (43.57)	0.019	127.78 (24.37)	127.99 (40.63)	0.957	127.78 (23.77)	127.99 (42.19)	0.955
Hematocrit	0.46 (1.52)	0.41 (0.52)	0.408	0.45 (1.26)	0.33 (0.10)	0.452	0.45 (1.28)	0.39 (0.15)	0.632	0.45 (1.29)	0.40 (0.23)	0.652
BUN (mmol/L)	4.70 (4.51)	7.06 (6.74)	< 0.001	5.35 (5.16)	12.37 (10.53)	< 0.001	5.27 (5.01)	10.40 (9.88)	< 0.001	4.93 (4.32)	13.50 (10.68)	< 0.001
Creatinine (μmol/L)	77.41 (35.51)	109.02 (99.68)	< 0.001	85.73 (60.61)	191.88 (159.04)	< 0.001	84.88 (58.86)	156.95 (140.72)	< 0.001	76.51 (28.59)	244.83 (163.11)	< 0.001
Total bilirubin (μmol/L)	38.25 (67.41)	41.66 (52.51)	0.277	38.41 (59.80)	72.16 (107.79)	0.023	39.54 (62.76)	39.40 (53.76)	0.983	39.07 (60.74)	45.55 (79.37)	0.274
Albumin (g/L)	37.78 (7.16)	34.19 (7.33)	< 0.001	36.69 (7.31)	28.97 (7.02)	< 0.001	36.6.69 (7.36)	32.31 (7.32)	< 0.001	36.76 (7.24)	32.04 (8.40)	< 0.001
AST (IU/L)	68.94 (113.56)	104.17 (293.67)	0.004	76.66 (121.03)	244.29 (894.16)	0.145	82.42 (206.45)	76.95 (82.21)	0.794	76.34 (120.21)	158.44 (618.75)	0.151
Calcium (mmol/L)	2.20 (0.24)	2.00 (0.33)	< 0.001	2.14 (0.27)	1.85 (0.63)	0.001	2.14 (0.27)	1.89 (0.48)	< 0.001	2.14 (0.27)	1.90 (0.44)	< 0.001
Blood glucose (mmol/L)	7.41 (3.84)	10.21 (5.37)	< 0.001	8.25 (4.47)	13.33 (7.78)	0.003	8.20 (4.40)	11.54 (7.09)	0.001	8.12 (4.26)	12.59 (7.69)	< 0.001
LDH (IU/L)	234.9 (104.21)	420.76 (235.22)	< 0.001	305.78 (186.17)	476.00 (326.16)	0.003	298.31 (177.79)	493.86 (291.49)	< 0.001	298.65 (176.75)	475.35 (300.05)	< 0.001
Triglycerides (mmol/L)	2.89 (4.37)	4.5 (6.08)	< 0.001	3.51 (5.12)	3.99 (6.52)	0.558	3.38 (5.00)	5.60 (6.88)	< 0.006	3.27 (4.79)	6.7 (8.06)	< 0.001
CRP (mg/L)	78.53 (58.34)	138.28 (60.29)	< 0.001	109.77 (66.56)	128.77 (62.19)	0.036	106.83 (65.32)	143.21 (66.71)	< 0.001	107.57 (66.88)	134.55 (58.07)	< 0.001
Ranson score	0.67 (0.77)	2.57 (1.37)	< 0.001	1.34 (1.36)	2.41 (1.58)	< 0.001	1.29 (1.33)	2.64 (1.46)	< 0.001	1.29 (1.32)	2.45 (1.61)	< 0.001
Glasgow score	0.48 (0.69)	2.24 (1.25)	< 0.001	1.09 (1.23)	2.39 (1.53)	< 0.001	1.05 (1.22)	2.45 (1.20)	< 0.001	1.04 (1.20)	2.28 (1.21)	< 0.001
BISAP	0.6 (0.72)	1.95 (1.1)	< 0.001	1.05 (1.06)	2.42 (1.25)	< 0.001	1.01 (1.04)	2.49 (1.00)	< 0.001	1.00 (1.02)	2.27 (1.20)	< 0.001
PASS	105.51 (52.27)	172.05 (81.08)	< 0.001	125.56 (66.82)	253.64 (94.44)	< 0.001	120.82 61.05)	273.19 (76.21)	< 0.001	120.73 (61.42)	243.11 (91.49)	< 0.001
CSSS	0.55 (0.78)	2.12 (1.50)	< 0.001	1.08 (1.29)	2.62 (1.69)	< 0.001	1.01 (1.22)	2.98 (1.65)	< 0.001	0.99 (1.19)	2.89 (1.66)	< 0.001

*ARDS* Acute respiratory distress syndrome, *ARF* Acute renal failure, *AP* Acute pancreatitis, *BMI* Body-mass index, *T2DM* type-2 diabetes mellitus; *WBC* White blood cell count, *BUN* Blood urea nitrogen, *CRP* C-reactive protein; AST: aspartate transaminase, *LDH* Lactate dehydrogenase, *BISAP* Bedside index of severity in acute pancreatitis, *PASS* Pancreatic activity scoring system, *CSSS* Chinese simple scoring system. *P* < 0.05 was accepted as statistically significant

### Value of biomarkers in predicting severity, mortality, and organ failure

In the multivariate analysis, white blood cell count (WBC), serum albumin, lactate dehydrogenase (LDH), calcium, glucose, and C-reactive protein (CRP) predicted the severity of AP. Their ORs for predicting severe AP were 1.110 (95% CI, 1.040–1.184), 0.940 (95% CI, 0.894–0.989), 1.004 (95% CI, 1.002–1.006), 0.196 (95% CI, 0.065–0.592), 1.081 (95% CI, 1.016–1.150), and 1.007 (95% CI, 1.003–1.012), respectively. Serum total bilirubin was found to be an independent predictor of mortality (OR, 1.013; 95% CI, 1.004–1.023). For predicting organ failure, BMI, WBC and serum calcium were independent variables for ARDS, while blood urea nitrogen and serum triglyceride were independent variables for ARF. However, among them only serum calcium showed a better OR value than other variables (Table 4).

**Table 4:** Multivariate analysis of factors predicting severity, mortality, ARDS, and ARF in AP

Characteristic	Severity		Mortality		ARDS		ARF	
	OR (95% CI)	*P*	OR (95% CI)	*P*	OR (95% CI)	*P*	OR (95% CI)	*P*
Age	0.994 (0.975–1.014)	0.575	1.023 (0.97–-1.069)	0.308	–	–	–	–
Male gender	–	–	–	–	–	–	0.731 (0.101–5.295)	0.731
BMI, kg/m^2^	0.985 (0.919–1.055)	0.66	–	–	1.139 (1.022–1.271)	0.019	1.125 (0.996–1.269)	0.057
Etiology								
Gallstones	1.256 (0.635–2.487)	0.512	0.255 (0.036–1.826)	0.174	1.794 (0.620–5.193)	0.281	0.974 (0.197–4.821)	0.974
Alcoholism	1.416 (0.526–3.808)	0.491	–	–	0.378 (0.074–1.923)	0.241	0.844 (0.153–4.649)	0.846
Hypertriglyceridemia							0.365 (0.065–2.036)	0.25
Smoker							0.996 (0.285–3.488)	0.995
Alcohol intake history	0.862 (0.467–1.590)	0.634	–	–	1.956 (0.657–5.827)	0.228	3.613 (0.810–16.122)	0.092
Comorbidities								
Hyperlipidemia	–	–	–	–	–	–	1.501 (0.529–4.26)	0.446
T2DM	–	–	–	–	–	–	0.999 (0.363–2.749)	0.998
WBC (*10^9^/L)	1.110 (1.040–1.184)	0.002	0.946 (0.819–1.094)	0.456	1.135 (1.048–1.23)	0.002	0.946 (0.839–1.067)	0.368
Hemoglobin (g/L)	–	–	1.023 (0.994–1.052)	0.118	–	–	–	–
BUN (mmol/L)	1.124 (0.974–1.297)	0.109	1.013 (0.914–1.122)	0.808	0.99 (0.917–1.069)	0.802	1.243 (1.097–1.408)	0.001
Creatinine (μmol/L)	1.005 (0.996–1.015)	0.268	1.006 (0.999–1.013)	0.105	1.002 (0.996–1.009)	0.484	–	–
Total bilirubin (μmol/L)	–	–	1.013 (1.004–1.023)	0.007	–	–	–	–
Albumin (g/L)	0.940 (0.894–0.989)	0.016	0.948 (0.833–1.079)	0.418	1.035 (0.978–1.095)	0.234	0.939 (0.854–1.032)	0.191
AST (IU/L)	1.002 (0.999–1.006)	0.18	–	–	–	–	–	–
Calcium (mmol/L)	0.196 (0.065–0.592)	0.004	0.882 (0.089–8.692)	0.914	0.042 (0.006–0.303)	0.002	1.205 (0.313–4.639)	0.786
Blood glucose (mmol/L)	1.081 (1.016–1.150)	0.014	1.023 (0.916–1.143)	0.686	1.021 (0.938–1.112)	0.624	1.054 (0.956–1.162)	0.294
LDH (IU/L)	1.004 (1.002–1.006)	< 0.001	1.003 (1.000–1.005)	0.061	1.000 (0.998–1.002)	0.785	1.000 (0.998–1.003)	0.781
Triglycerides (mmol/L)	1.022 (0.961–1.086)	0.486	–	–	0.943 (0.845–1.051)	0.287	1.119 (1.012–1.239)	0.029
CRP (mg/L)	1.007 (1.003–1.012)	0.002	0.999 (0.988–1.011)	0.844	1.002 (0.995–1.008)	0.409	1.000 (0.993–1.008)	0.926

*ARDS* Acute respiratory distress syndrome, *ARF* Acute renal failure, *AP* acute pancreatitis, *BMI* Body-mass index, *T2DM* Type-2 diabetes mellitus, *CRP* C-reactive protein, *AST* Aspartate transaminase, *BUN* Blood urea nitrogen, *LDH* Lactate dehydrogenase, *BISAP* Bedside index of severity in acute pancreatitis, *PASS* Pancreatic activity scoring system, *CSSS* Chinese simple scoring system, *WBC* White blood cell count, *OR* Odds ratio, *CI* Confidence interval. *P* < 0.05 was accepted as statistically significant

### Value of scoring Systems in Predicting Severity, mortality, and organ failure

**Fig. 1:**
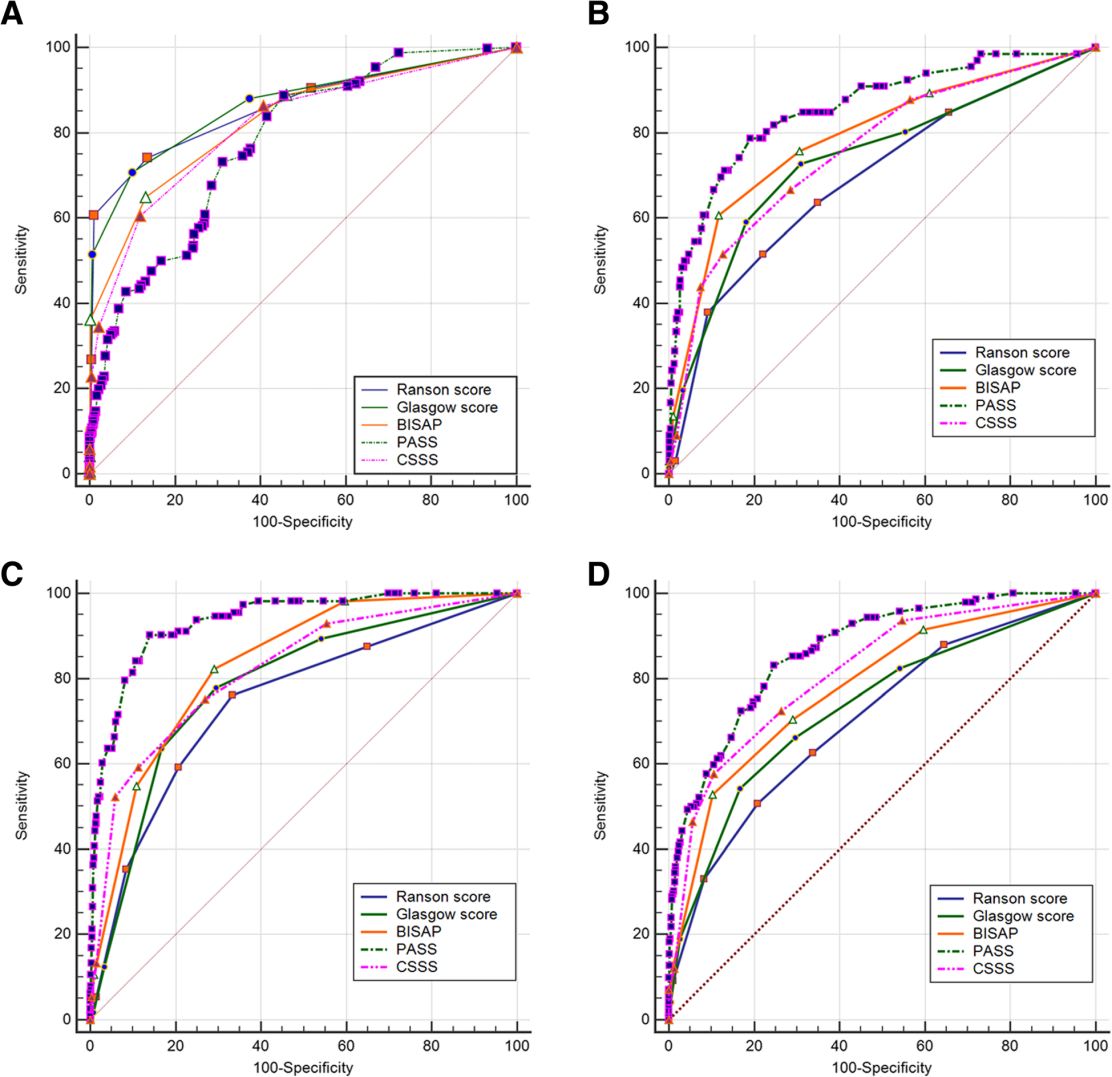
**a** Receiver operating characteristic curves of scoring systems to predict severe AP. **b** with AP. **c** Receiver operating characteristic curves of scoring systems to predict ARDS in patients with AP. **d** Receiver operating characteristic curves of scoring systems to predict ARF in patients with AP

For severe AP prediction, the ROC curve indicated an AUC of 0.861 for RS, 0.865 for GS, 0.829 for BISAP, 0.778 for PASS, and 0.816 for CSSS. The cutoffs used were as follow: RS, at least 2; GS, at least 2; BISAP, at least 2; PASS, at least 90; and CSSS, at least 2 (Table 5, Fig. 1a). For mortality prediction, the AUCs of the scoring systems were as follow: 0.693 for RS, 0.736 for GS, 0.789 for BISAP, 0.858 for PASS, and 0.759 for CSSS. The cutoffs of the scoring systems for mortality prediction were as follow: RS, at least 3; GS, at least 2; BISAP, at least 3; PASS, at least 190; and CSSS, at least 3 (Table 5, Fig. 1b). For ARDS prediction, the AUCs of scoring systems were as follow: 0.745 for RS, 0.784 for GS, 0.834 for BISAP, 0.936 for PASS, and 0.820 for CSSS. The cutoffs for RS, GS, BISAP, and CSSS were all at least 2, and the cutoff for PASS was at least 195 (Table 5, Fig. 1c). For ARF prediction, the AUCs of the scoring systems were as follow: 0.707 for RS, 0.734 for GS, 0.781 for BISAP, 0.868 for PASS, and 0.816 for CSSS. The cutoffs for RS, GS, BISAP, and CSSS were all at least 3, and the cutoff for PASS was at least 65 (Table 5, Fig. 1d).

**Table 5:** Effectiveness of scoring systems for predicting severity, mortality, ARDS, and ARF in AP

	Cutoff	AUC (95% CI)	Sensitivity (95% CI)	Specificity (95% CI)	PPV (95% CI)	NPV (95% CI)
Severity						
Ranson score	≥2	0.861 (0.844–0.876)	0.741 (0.707–0.774)	0.864 (0.843–0.883)	0.762 (0.728–0.794)	0.850 (0.828–0.870)
Glasgow score	≥ 2	0.865 (0.849–0.881)	0.708 (0.672–0.742)	0.900 (0.882–0.917)	0.807 (0.773–0.838)	0.840 (0.818–0.860)
BISAP	≥ 2	0.829 (0.811–0.846)	0.649 (0.612–0.685)	0.869 (0.848–0.887)	0.744 (0.707–0.778)	0.808 (0.785–0.830)
PASS	≥ 90	0.778 (0.759–0.797)	0.889 (0.863–0.912)	0.545 (0.516–0.574)	0.534 (0.505–0.564)	0.893 (0.868–0.915)
CSSS	≥ 2	0.816 (0.797–0.833)	0.605 (0.568–0.642)	0.894 (0.876–0.910)	0.750 (0.712–0.786)	0.812 (0.791–0.832)
Mortality						
Ranson score	≥3	0.693 (0.671–0.714)	0.515 (0.389–0.640)	0.976 (0.967–0.983)	0.500 (0.376–0.624)	0.978 (0.968–0.985)
Glasgow score	≥ 2	0.736 (0.715–0.756)	0.727 (0.604–0.830)	0.690 (0.668–0.712)	0.080 (0.060–0.105)	0.986 (0.977–0.991)
BISAP	≥ 3	0.789 (0.770–0.807)	0.606 (0.478–0.724)	0.882 (0.866–0.897)	0.160 (0.117–0.211)	0.984 (0.976–0.989)
PASS	≥ 190	0.858 (0.841–0.874)	0.788 (0.670–0.879)	0.809 (0.790–0.827)	0.133 (0.101–0.170)	0.990 (0.984–0.995)
CSSS	≥ 3	0.759 (0.738–0.778)	0.515 (0.389–0.640)	0.872 (0.856–0.887)	0.130 (0.092–0.177)	0.980 (0.972–0.986)
ARDS						
Ranson score	≥2	0.745 (0.725–0.765)	0.761 (0.672–0.836)	0.666 (0.644–0.689)	0.129 (0.105–0.157)	0.977 (0.967–0.985)
Glasgow score	≥2	0.784 (0.764–0.802)	0.779 (0.691–0.851)	0.705 (0.683–0.726)	0.147 (0.119–0.178)	0.980 (0.971–0.987)
BISAP	≥2	0.834 (0.816–0.851)	0.823 (0.740–0.888)	0.710 (0.688–0.731)	0.156 (0.127–0.187)	0.984 (0.975–0.990)
PASS	≥ 195	0.936 (0.924–0.946)	0.903 (0.833–0.950)	0.860 (0.843–0.876)	0.296 (0.248–0.347)	0.993 (0.987–0.996)
CSSS	≥ 2	0.820 (0.802–0.838)	0.752 (0.662–0.829)	0.731 (0.709–0.752)	0.154 (0.125–0.187)	0.978 (0.969–0.986)
ARF						
Ranson score	≥ 3	0.707 (0.686–0.728)	0.507 (0.422–0.592)	0.792 (0.772–0.811)	0.169 (0.134–0.208)	0.951 (0.938–0.961)
Glasgow score	≥ 3	0.734 (0.711–0.752)	0.542 (0.457–0.626)	0.857 (0.841–0.872)	0.213 (0.172–0.259)	0.963 (0.954–0.972)
BISAP	≥ 3	0.781 (0.761–0.800)	0.346 (0.283–0.413)	0.897 (0.882–0.911)	0.300 (0.244–0.361)	0.915 (0.901–0.928)
PASS	≥ 165	0.868 (0.852–0.883)	0.831 (0.759–0.889)	0.754 (0.733–0.774)	0.219 (0.185–0.257)	0.982 (0.973–0.988)
CSSS	≥ 3	0.816 (0.798–0.834)	0.578 (0.492–0.660)	0.895 (0.879–0.909)	0.313 (0.257–0.373)	0.962 (0.952–0.971)

*ARDS* Acute respiratory distress syndrome, *ARF* Acute renal failure, *AP* Acute pancreatitis, *AUC* Area under the curve, *PPV* Positive predictive value, *NPV* negative predictive value; *BUN* Blood urea nitrogen; *BISAP* Bedside index of severity in acute pancreatitis, *PASS* Pancreatic activity scoring system, *CSSS* Chinese simple scoring system, *CI* confidence interval

## Discussion

In the present study, BMI was an independent factor for the development of ARDS in AP patients, which is consistent with the result of a meta-analysis, that demonstrated that obesity was an important risk factor for the development of ARDS [26]. Studies have shown that patients who are obese have higher levels of circulating neutrophils [27] and blood cytokines [28], and have low-grade chronic inflammation triggered by obesity [29]. Moreover, innate immune cell activation and endothelial injury in the pulmonary microvasculature are major contributors to increased cell permeability and pulmonary edema in obese patients [30, 31].

This study revealed that serum Ca^2+^ showed good ORs for severity and ARDS prediction. Abnormal regulation of Ca^2+^ signals acts as a crucial trigger in the pathogenesis of AP [32]. A study has shown that hypocalcemia is an independent risk factor of severe AP and for respiratory failure in AP [33]. According to the present study, the WBC predicted the development of severe AP and ARDS. Furthermore, serum albumin, glucose, LDH, and CRP were also predictive factors for severe AP. These biomarkers are commonly used factors to predict severe AP. In terms of mortality prediction, the multivariate analysis identified that an increase in serum total bilirubin was a risk factor. Although few studies have reported a definite relationship between total bilirubin and mortality in AP, some studies have found that the albumin-bilirubin score has a high predictive capacity for in-hospital mortality or prognosis in patients with critical diseases such as acute upper gastrointestinal bleeding due to liver cirrhosis [34], post-operative hepatic carcinoma [35, 36], and AP [37]. Moreover, the present study showed that the elevation of serum triglycerides was a risk factor for ARF in AP patients, which is consistent with the findings of a meta-analysis reported in 2018 [38].

RS, GS, and BISAP showed high accuracy in predicting the severity rather than the outcomes of AP in the present study. RS and GS predicted the severity and 3 outcomes of AP equally well, which was probably due to the similar parameters they share. Although simple, these scores are not repeatable. According to this study, BISAP was inferior to both RS and GS in predicting severity, which is consistent with the findings of other prospective studies [39, 40]. This is because the items in RS and GS cover more systems than those in BISAP. Nevertheless, BISAP was superior to RS and GS in predicting mortality in the present study. Hall et al. also found that RS and GS were not good indicators of mortality in AP [41]. BISAP was also better than RS and GS at predicting ARDS and ARF, possibly because it is based on 3 important items that are related to the renal and respiratory systems, namely, blood urea nitrogen (BUN), systemic inflammatory response syndrome (SIRS), and pleural effusion.

PASS is a system that assesses the activity of AP at any time during hospitalization. It contains not only objective items (organ failure and SIRS), but also subjective items (abdominal pain, morphine usage and ability to tolerate solid diet). The repeatable items make it available to be used at any time during hospitalization. A prospective study [18] demonstrated that a cutoff PASS score of > 140 on admission was associated with an AUC of 0.71 for predicting severe AP. The present study found a similar AUC for PASS for severe AP prediction. As the center in which this study was conducted rarely uses morphine to relieve abdominal pain in patients with AP, the cutoff for severity prediction was only 90. In the present study, PASS scores best predicted mortality and organ failure, especially ARDS prediction. This is because PASS contains organ failure items. However, its subjective items (such as abdominal pain, morphine usage and ability to tolerate solid diet) make it inferior to other scores in severity prediction. Thus, no study has reported the predictive ability of PASS for the outcomes of AP.

Four biomarkers, heart rate, and pancreatic imaging findings are included in CSSS. According to the present study, the AUCs of CSSS for severity and mortality prediction were 0.834 and 0.838, respectively. The cutoff points were 4 for severity and 6 for mortality. However, the AUCs and cutoff points in this study are smaller than those reported in the previous study [20], which is probably attributable to the larger sample size of the current study. In the present study, CSSS showed nearly the same ability in predicting the 4 outcomes of AP, and it shared nearly equal capacity with BISAP for predicting the outcomes of AP, which indicates that CSSS is a promising scoring system. However, no study evaluating CSSS was found. Hence, studies with larger sample size and prospective designed are required to verify the efficiency of this new scoring system.

### Study strengths and limitations

The strengths of the present study are that it compared both conventional and novel scoring systems as well as biomarkers in a large sample of Chinese patients for the prediction of the severity and outcomes of AP.

This study does have some limitations. First, this was a retrospective single-center study. Second, there was diversity in the period between the onset of AP and admission. This probably resulted in heterogeneity in the timings of score calculations and biochemical marker measurements.

## Conclusion

RS and GS predicted severity better than mortality and organ failure, while PASS predicted mortality and organ failure better. As a novel scoring system, PASS has potential, but some of its items are not that suitable for Chinese medical centers. BISAP and CSSS performed equally well in severity and outcome prediction.

## Data Availability

All data used in this study are available from the corresponding author.

## Abbreviations

ARF: Acute renal failure
ARDS: Acute respiratory distress syndrome
RS: Ranson score
GS: Glasgow score
APACHE: Acute physiology and chronic health evaluation
BISAP: Bedside index of severity in acute pancreatitis
PASS: Pancreatic activity scoring system
CSSS: Chinese simple scoring system
AUC: Area under the curve
ROC: Receiver operating characteristic
BMI: Body-mass index
T2DM: Type-2 diabetes mellitus
WBC: White blood cell count
BUN: Blood urea nitrogen
SIRS: Systemic inflammatory response syndrome
LDH: Lactate dehydrogenase
CRP: C-reactive protein
AST: Aspartate transaminase
